# SARS-CoV-2-specific immune response in COVID-19 convalescent individuals

**DOI:** 10.1038/s41392-021-00686-1

**Published:** 2021-07-07

**Authors:** Yunbao Pan, Xianghu Jiang, Liu Yang, Liangjun Chen, Xiaojiao Zeng, Guohong Liu, Yueting Tang, Chungen Qian, Xinming Wang, Fangming Cheng, Jun Lin, Xinghuan Wang, Yirong Li

**Affiliations:** 1 Department of Laboratory Medicine, Zhongnan Hospital of Wuhan University, Wuhan University, Wuhan, Hubei China; 2 Wuhan Research Center for Infectious Diseases and Cancer, Chinese Academy of Medical Sciences, Wuhan, China; 3 Department of Radiology, Zhongnan Hospital of Wuhan University, Wuhan University, Wuhan, China; 4 The Key Laboratory for Biomedical Photonics of MOE at Wuhan National Laboratory for Optoelectronics – Hubei Bioinformatics & Molecular Imaging Key Laboratory, Systems Biology Theme, Department of Biomedical Engineering, College of Life Science and Technology, Huazhong University of Science and Technology, Wuhan, Hubei China; 5 Reagent R&D Center, Autobio Diagnostics Co., Ltd, Zhengzhou, Henan China; 6 Reagent R&D Center, Shenzhen YHLO Biotech Co., Ltd, Shenzhen, Guangdong China; 7 Department of Gastroenterology, Zhongnan Hospital of Wuhan University, Wuhan University, Wuhan, China; 8 Center for Evidence-Based and Translational Medicine, Zhongnan Hospital of Wuhan University, Wuhan University, Wuhan, China; 9 Department of Urology, Zhongnan Hospital of Wuhan University, Wuhan University, Wuhan, China

**Keywords:** Infectious diseases, Immunological disorders

## Abstract

We collected blood from coronavirus disease 2019 (COVID-19) convalescent individuals and investigated SARS-CoV-2-specific humoral and cellular immunity in these discharged patients. Follow-up analysis in a cohort of 171 patients at 4–11 months after the onset revealed high levels of IgG antibodies. A total of 78.1% (164/210) of the specimens tested positive for neutralizing antibody (NAb). SARS-CoV-2 antigen peptide pools-stimulated-IL-2 and -IFN-γ response can distinguish COVID-19 convalescent individuals from healthy donors. Interestingly, NAb survival was significantly affected by the antigen peptide pools-stimulated-IL-2 response, -IL-8 response, and -IFN-γ response. The antigen peptide pools-activated CD8+ T cell counts were correlated with NAb. The antigen peptide pools-activated natural killer (NK) cell counts in convalescent individuals were correlated with NAb and disease severity. Our data suggested that the development of NAb is associated with the activation of T cells and NK cells. Our work provides a basis for further analysis of the protective immunity to SARS-CoV-2 and for understanding the pathogenesis of COVID-19. It also has implications for the development of an effective vaccine for SARS-CoV-2 infection.

## Introduction

Severe acute respiratory syndrome coronavirus 2 (SARS-CoV-2) related coronavirus disease (COVID-19) is a respiratory transmissible disease that can cause death from severe illness. SARS-CoV-2 has the same receptor and host cell as SARS-CoV. Many disease models have been established to investigate the infection, immunogenicity, and pathogenesis of SARS-CoV-2. Based on previous knowledge of other coronaviruses, various factors and pathways have been identified and found to be promising potential therapeutic targets.^1,2^ However, developing effective treatments requires a more comprehensive understanding, which requires better molecular detail at different stages of viral reproduction and disease progression in host cells. In the early and mild stages of infection, the virus remains confined to the upper respiratory tract, causing a low level of innate immune response. This asymptomatic state lasts for a few days before the virus spreads to the catheter and terminal airways. At this stage of the disease, an optimal but controlled adaptive and innate immune response will help fight infection. Successful elimination of the virus from recovered patients indicates the presence of adequate adaptive immune cells as well as immune regulatory molecules and neutralizing antibodies.^1,3^ However, impairment of the adaptive immune response at this stage, along with innate immune system hyperactivation, can cause severe disease symptoms in COVID-19 patients.^4^ Histopathology data from deceased patients demonstrate adaptive immune dysfunction and an enhanced pro-inflammatory response, with inflammatory cell infiltration into the lungs. In addition, disease severity has been found to be positively associated with increased levels of pro-inflammatory interleukin-6 (IL-6) and neutrophil lymphocyte ratio.^5,6^ Patients with COVID-19 may develop acute respiratory distress syndrome (ARDS) from excessive inflammation and lymphocytopenia.^7^ These changes lead to cell death and tissue destruction, resulting in airway collapse, multiple organ failure, and death in 67–85% of intensive care unit (ICU) patients.^4,8^

Clinically, the host immune system is involved in the pathogenesis of the disease.^9,10^ The protective and persistent immune response to viral infection usually arise from the combined actions of lymphocytes: B cells (responsible for humoral antibody immunity) and T cells (responsible for cellular immunity and helping B cell responses).^11,12^ B cells produce detectable IgM, IgG, and IgA antibodies, along with smaller amounts of IgD and IgE. For SARS-CoV-2, the focus is mainly on IgM, IgG, and IgA antibodies that can neutralise the virus by binding to the spike and other membrane proteins and thus preventing infection.^11,13^ A few studies have focused on the immune response to SARS-CoV-2 infection, especially on the characteristics of adaptive immune response. A high titer of the IgG antibody has been reported in 8 newly discharged patients and 6 patients at 2 weeks after discharge. The neutralizing antibody (NAb) is also associated with the number of specific T cells.^14^ However, the study did not distinguish between CD4+ and CD8+ T cell responses. Based on these reports, we can infer that the antibody response of some COVID-19 patients may not last long, which poses a challenge for antibody-based therapy and vaccine research, these data warn of the possibility of reinfection. However, longitudinal studies with larger cohort sizes and longer time frames are needed to discover the persistence of the SARS-CoV-2-specific antibody response.

In this study, we collected blood from virus-free COVID-19 convalescent individuals to explore the immune response of host cells, and analyzed their SARS-CoV-2-specific antibody and the response of CD4+, CD8+, and natural killer (NK) cells to SARS-CoV-2 antigen peptide pools. The virus-specific lymphocytes and their association with NAbs were also detected in patients with COVID-19. We established the immune trajectories of COVID-19 patients who successfully cleared the virus, as well as the temporal effects of innate and adaptive immune systems.

## Results

### Patients’ information

A total of 212 samples from 171 COVID-19 convalescent individuals, with a median age of 52 years (range from 23 to 83 years) who were hospitalized in Zhongnan Hospital of Wuhan University and who recovered from the SARS-CoV-2 infection (83 males vs. 88 females), were enrolled in the study. In 131 patients, the blood was collected once; in 39 patients, the blood was collected twice; and in 1 patient, the blood was collected thrice. Their clinical and pathological characteristics are shown in Supplemental Table 1. COVID-19 convalescent individuals were recruited in three time periods: July 15, 2020 solstice July 31, 2020, September 7, 2020 solstice September 23, 2020, November 11, 2020 solstice December 10, 2020, and thus been classified into three categories (4–6, 7–8, and 9–11 months). The date of diagnosis of COVID-19 was defined as day zero in our follow-up. All convalescent individuals initially showed symptoms via computed tomography (CT) scan and were positive on SARS-CoV-2 nucleic acid testing. Thirty additional healthy donors were recruited in this study. The study was conducted with the consent of patients and was approved by the Ethics Committee of Zhongnan Hospital of Wuhan University.

### Detection of SARS-CoV-2-specific antibodies in COVID-19 convalescent individuals

**Fig. 1:**
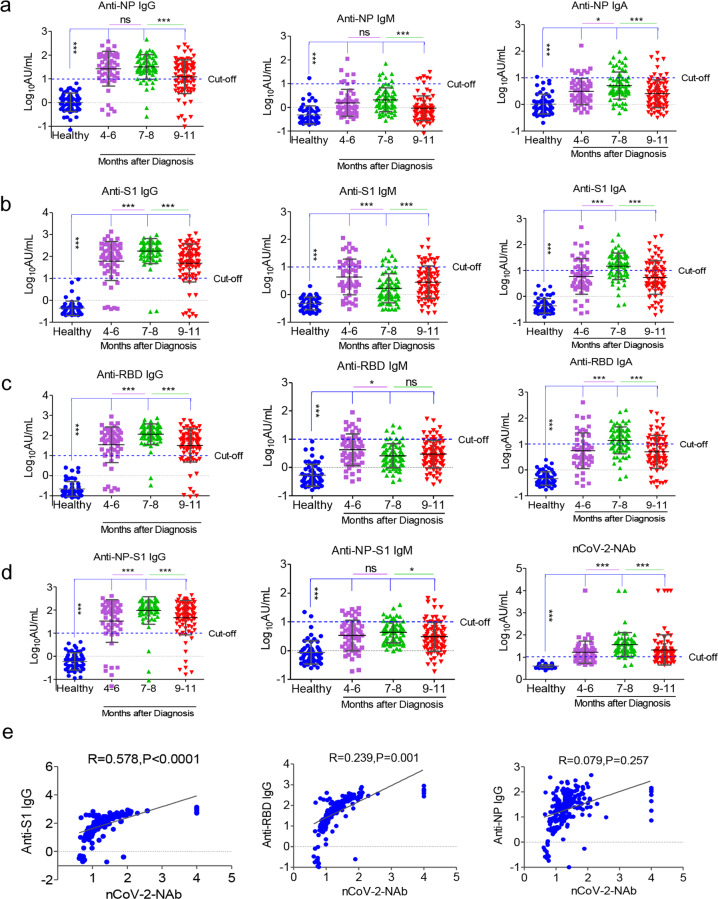
SARS-CoV-2-specific antibodies and NAb in COVID-19 convalescent individuals. NP- (**a**), S1- (**b**), RBD- (**c**), and NP-S1- (**d**) specific IgG, IgM, and IgA antibodies and NAb (**d**, right) were detected in the sera of COVID-19 convalescent individuals, compared with the healthy donor group. (**e**) NAbs were correlated with ant-S1 IgG, anti-RBD IgG, and anti-NP IgG. *P < 0.05, 0.05 < **P < 0.001, ***P < 0.001

Using sera from patients and healthy donors, IgA, IgM, and IgG antibodies against SARS-CoV-2 NP, S1, RBD, and NP-S1 antigens were detected. There was a significant antibody response in the patients’ sera (Fig. 1). NP-, S1-, RBD-, and NP-S1-specific IgA, IgM, and IgG antibodies were detected in the sera of COVID-19 convalescent individuals, compared with the healthy donor group. Anti-SARS-CoV-2 IgG antibodies were also more obviously observed than IgA and IgM antibodies in the follow-up patients when compared with healthy donors (Fig. 1). Overall, these findings suggest that COVID-19 patients show IgG, IgA, and IgM responses to SARS-CoV-2 proteins, especially NP, S1, RBD, and NP-S1, and they also suggest that infected patients can maintain their IgG level until at least 11 months after the onset of illness.

Besides, since RBD of the S protein has been shown to bind to the human angiotensin-converting enzyme 2 (ACE2),^15^ the existence of antibodies may suggest neutralization of SARS-CoV-2 infection. The iFlash-2019-nCoV Neutralization Antibody assay (NAb)　is a one-step competitive immunoassay using the direct chemiluminometric technique: SARS-CoV-2 NAb in the sample (if it exists) reacts with SARS-CoV-2 receptor-binding domain (RBD) antigen-coated paramagnetic microparticles to form a complex, and then an acridinium-ester-labeled ACE2 conjugate is added to competitively combine with the RBD-coated particles, which have not been neutralized by the NAb (if it exists) from the sample, and form another reaction mixture. An inverse relationship exists between the amount of SARS-CoV-2 NAb in the sample and the RLUs detected by the iFlash optical system. As shown in Fig. 1, 67.3% (37/55) of patients who were followed up for 4–6 months following the onset of illness had positive NAb, while 94.4% (67/71) and 71.4% (60/84) of patients had positive NAb within 7–8 months and 9–11 months after the diagnosis, respectively. As expected, NAbs were significantly correlated with anti-S1 IgG and anti-RBD IgG, but not with anti-NP IgG (Fig. 1e), suggesting that anti-S1 IgG and anti-RBD IgG may be the predictors of serum neutralizing ability in COVID-19 patients. These findings suggest that most of the COVID-19 convalescent individuals have serum neutralizing SARS-CoV-2 until at least 11 months following the onset of illness.

### Dynamic changes in lymphocyte subsets and cytokines in COVID-19 convalescent individuals

**Fig. 2:**
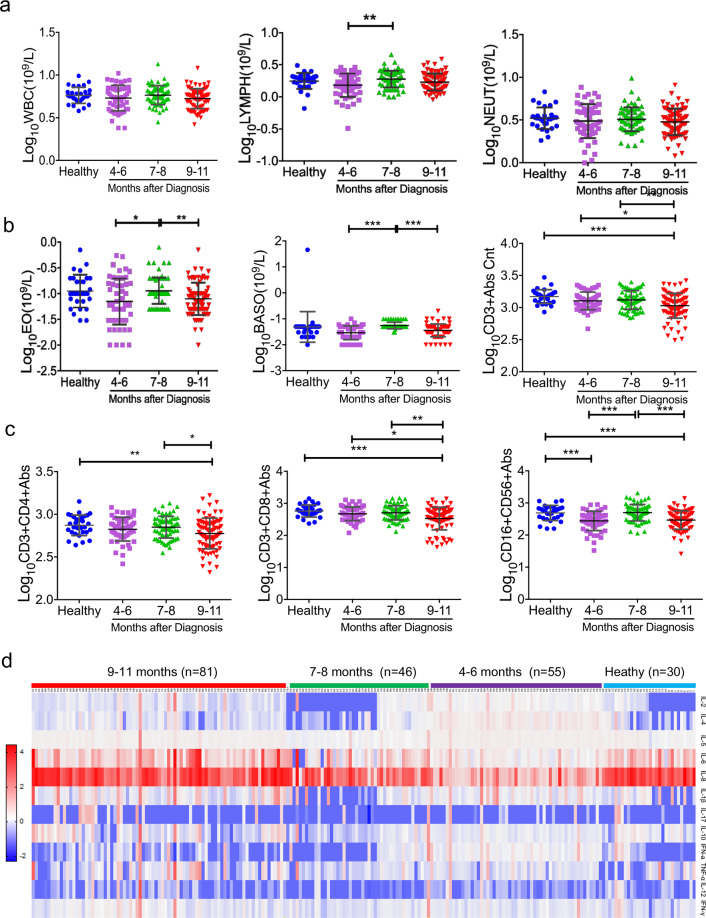
Dynamic changes in lymphocyte subsets and cytokines in COVID-19 convalescent individuals. The routine hematological examination was performed using standard methods in our hospital. Routine peripheral blood cells, including WBC, lymphocytes, neutrophils, eosinophils, and basophils, were analyzed. Lymphocyte subsets were analyzed in COVID-19 convalescent individuals (**a**–**c**). A total of 12 kinds of cytokines were analyzed in COVID-19 convalescent individuals by flow cytometry (**d**). Samples were organized by the time course after the symptom onset, which is indicated on the top side of the heat-map

In this study, we analyzed the dynamic changes in WBC, lymphocyte counts, B cell counts, NK cell counts, as well as in different lymphocyte subsets in COVID-19 convalescent individuals (Fig. 2a). The lymphocyte counts were markedly lower in the 4–6 months group compared with the convalescent individuals group at 7–8 months after the diagnosis. CD3+ T cells and CD8+ T cells were markedly lower in the 9–11 months group compared with those in the convalescent individuals group at 7–8 months after the diagnosis. NK (CD16+CD56+) cells were increased within 7–8 months and then they were decreased within 9–11 months. No significant differences were observed in WBC, CD4+ T cells, and B cell counts among the groups during the follow-up (Fig. 2). The proportion of patients with abnormal laboratory features are presented in Supplemental Table 1. A significantly higher proportion of COVID-19 convalescent individuals presented with reduced CD3+CD8+ cells, B cells, and NK cells.

In this study, we also analyzed the data of 12 kinds of cytokines in the convalescent individuals (Fig. 2d and Supplemental Fig. 2). The IL-4 level was decreased within 7–8 months and then it was increased within 9–11 months. There was a trend toward an increased frequency of IL-8 in the follow-up patients. No significant differences were observed in IL-2, IL-12, IFN-γ, IL-5, IL-6, IL-1β, IL-17, IL-10, IFN-α, and TNF-α among the groups during the follow-up (Fig. 2b and Supplemental Fig. 2). The proportion of patients with abnormal cytokine features is presented in Supplemental Table 2. A significantly higher proportion of COVID-19 convalescent individuals presented with increased IL-5, IL-6, and IL-1β levels.

**Fig. 3:**
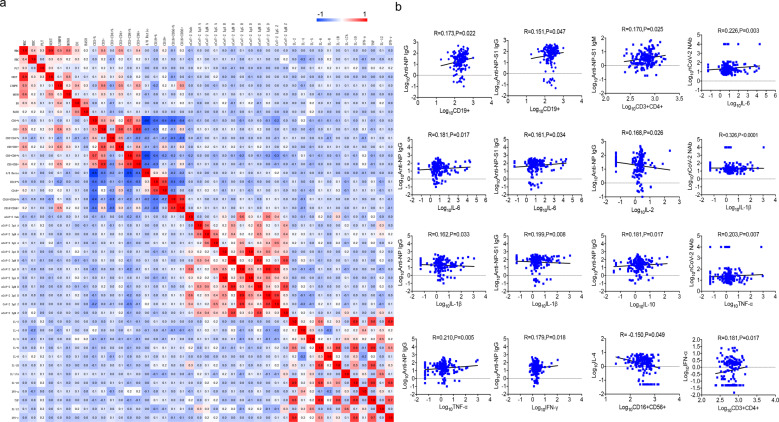
Correlation of antibodies with lymphocyte subsets and cytokines in COVID-19 convalescent individuals. Pearson correlation between different antibodies and lymphocyte subsets and cytokines. **a** CorHeatmap was used to color these correlations: blue indicates negative correlations, red indicates positive correlations, and the color intensity indicates the strength of the correlation. **b** Scatter diagrams were used to describe the correlation

As expected, B cell counts were significantly correlated with anti-NP IgG and anti-NP-S1 IgG, but not with NAb (Fig. 3). With respect to the NAb, it was correlated with IL-6, IL-1β, and TNF-α. These findings suggest that although normal lymphocyte subsets and cytokines were found in most of the convalescent individuals, they may be the predictors of serum neutralization capacity in COVID-19 patients.

### Cellular immune responses to SARS-CoV-2 in COVID-19 convalescent individuals

**Fig. 4:**
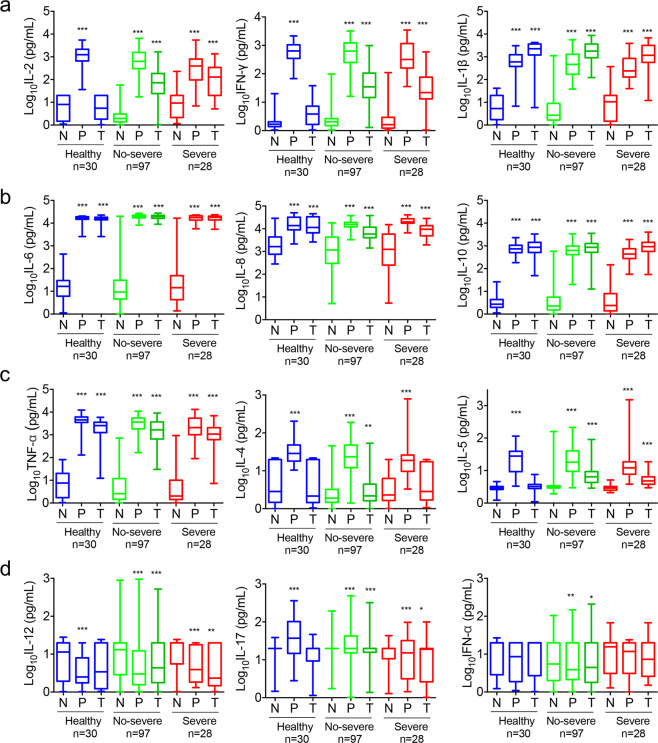
Cytokine responses to SARS-CoV-2 in COVID-19 convalescent individuals. Whole blood from COVID-19 convalescent individuals and healthy donors was treated with recombinant antigen peptide pools (NP, S1, S2, and S-RBD) and PHA control overnight, followed by cytokine analysis. (**a**–**d**) Responses of 12 kinds of cytokines were described by a histogram. N, negative control, without treatment. P, PHA treatment. T, antigen peptide pools (NP, S1, S2, and S-RBD) treatment. Severe, individuals in the convalescent phase after severe disease

To assess virus-specific cellular immunity, we treated whole blood with recombinant antigen peptide pools (NP, S1, S2, and RBD) and phytohemagglutinin (PHA) control, followed by cytokine analysis. We decided to use whole blood instead of peripheral blood mononuclear cells (PBMCs) to detect the secretion of cytokines to improve the applicability in clinical practice. The results were considered positive if there was at least a twofold increase in the treated subjects than in the untreated subjects (negative control) and were above the normal range of the concentration of cytokines. As shown in Fig. 4, the recombinant antigen peptide pools developed strong immune responses by increasing IL-1β, IL-6, IL-8, IL-10, and TNF-α in both healthy donors and patients. Of note, compared with healthy donors, the concentrations of recombinant antigen peptide pools induced-IL-2 and -IFN-γ in convalescent individuals were much higher, suggesting that IL-2 and IFN-γ had induced SARS-CoV-2-specific responses. Interestingly SARS-CoV-2 seronegative healthy donors also showed the presence of antigen peptide pools reactive IL-1β, IL-6, IL-8, IL-10, and TNF-α, suggesting the presence of SARS-CoV-2-nonspecific responses. At the same time, PHA control stimulated most of the cytokines, but it did not stimulate the secretion of IL-12, IL-17, or IFN-α.

**Fig. 5:**
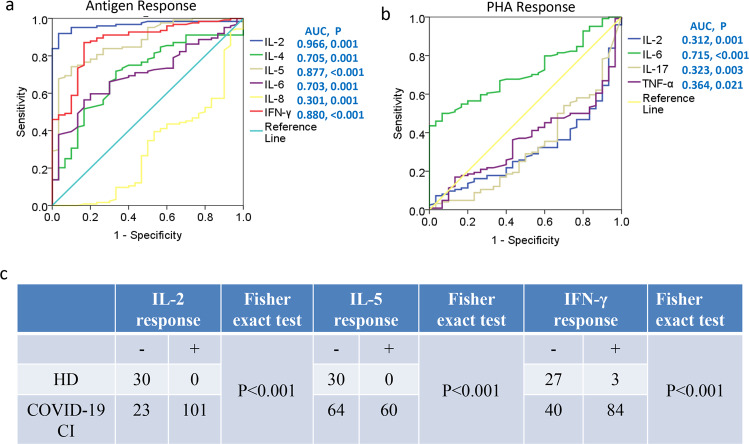
SARS-CoV-2 antigen-activated cytokine responses distinguished COVID-19 convalescent individuals from healthy donors. Receiver operator characteristic curves for cytokines activated by the antigen (**a**), and activated by PHA (**b**), comparison of data for COVID-19 convalescent individuals and healthy donors. **c** SARS-CoV-2 antigen-activated IL-2, IL-5, and IFN-γ were associated with COVID-19 convalescent individuals. ROC, Receiver operating characteristic, AUC, area under curve, HD, healthy donor, CI, convalescent individual.

To investigate the diagnostic significance of activated cytokines in COVID-19 convalescent individuals, an ROC curve analysis was performed. As shown in Fig. 5a, the AUC values for antigen peptide pools stimulated-IL-2 and -IFN-γ were 0.966 and 0.88, respectively. The predictive value of the mix stimulated-cytokines for COVID-19 convalescent individuals was superior to that of the PHA stimulated-cytokines (Fig. 5b). Furthermore, COVID-19 convalescent individuals showed accompanying antigen peptide pools stimulated-IL-2 response, -IFN-γ response, and -IL-5 response (Fig. 5c). These data suggested that antigen peptide pools stimulated-IL-2 and -IFN-γ could distinguish COVID-19 convalescent individuals from healthy donors.

### Associations of activated cytokines with antibody survival

**Fig. 6:**
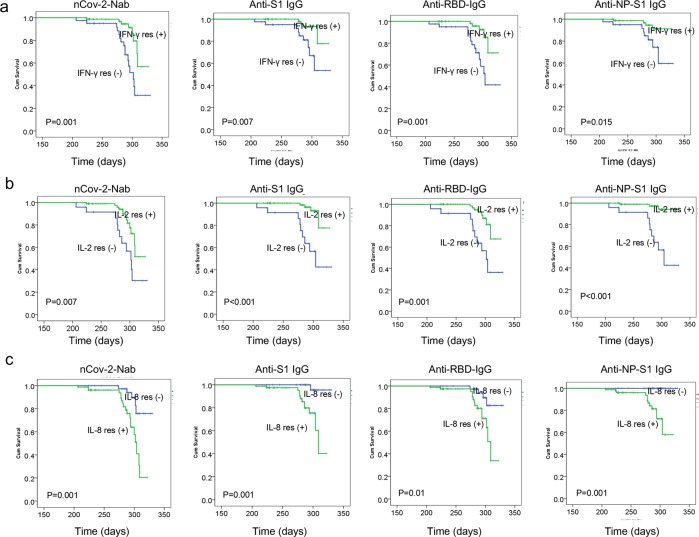
SARS-CoV-2 antigen-activated cytokine responses associated with antibody survival. Kaplan–Meier analysis of the association of antigen-activated IFN-γ responses and antibody survival (**a**), the association of antigen-activated IL-2 responses and antibody survival (**b**), and the association of antigen-activated IL-8 responses and antibody survival (**c**)

In multivariate Cox regression analysis, NAb survival was significantly affected by the antigen peptide pools stimulated-IL-2 response, -IL-8 response, and -IFN-γ response (Supplemental Table 3). By Kaplan–Meier analysis and the log-rank test, antigen peptide pools stimulated-IL-2 and -IFN-γ positive responses were associated with significantly better NAb, anti-S1 IgG, anti-RBD IgG, and anti-NP-S1 IgG survival in COVID-19 convalescent individuals, while the antigen peptide pools stimulated-IL-8 positive response was associated with significantly worse antibody survival (Fig. 6).

**Fig. 7:**
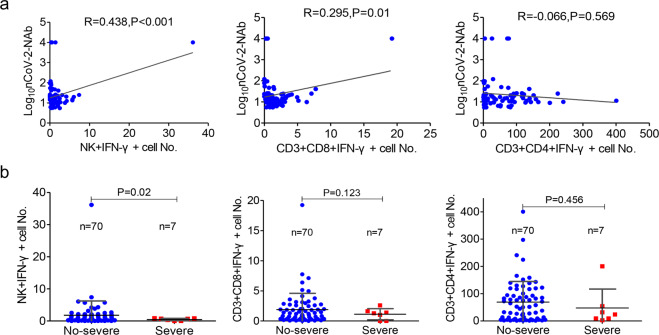
SARS-CoV-2 antigen-activated cellular immune responses associated with antibodies. **a** Correlation between the neutralizing antibody (Nab) and the number of antigen-specific NK cells, CD3+CD8+ cells, and CD3+CD4+ cells. **b** The number of antigen-specific NK cells in the severe group was much lesser than that in the non-severe group

In addition, in order to simultaneously detect IFN-γ secretion in CD4+, CD8+ T cells, and NK cells in the same tube, we used six fluorescent antibodies to label different lymphocyte subsets and the final results could be seen in the flow cytometry analysis template (Supplemental Fig. 3). We analyzed the effects of antigen peptide pools and PMA/ionomycin on the secretion of IFN-γ by T cells and NK cells. The data showed that PMA/ionomycin could induce simultaneous secretion of IFN-γ from CD4+, CD8+ T cells, and NK cells. More interestingly, on combining all 77 patients in our analysis, there was a significant correlation between the NAb and the number of antigen-specific NK cells and CD3+CD8+ cells (Fig. 7a), indicating that the development of NAbs may be associated with the activation of antiviral T cells and NK cells. In addition, the number of antigen-specific NK cells in the severe group was much smaller than that in the non-severe group (Fig. 7b). Therefore, effective elimination of the virus may require a synergistic humoral and cellular immune response.

## Discussion

In this study, we described SARS-CoV-2-specific humoral and cellular immunity in COVID-19 convalescent individuals. NAbs were significantly correlated with the number of SARS-CoV-2-specific T and NK cells. Our work indicate that B, T, and NK cells are involved in immune-mediated protection against viral infection. Our findings provided a basis for further analysis of protective immunity of SARS-CoV-2 and for understanding the pathogenesis of COVID-19. It also makes sense to design an effective vaccine to protect against and treat SARS-CoV-2 infection.

Understanding the duration of antibody responses to SARS-CoV-2 will be the key to continued prevention of reinfection. Of all human-infectious coronaviruses, the SARS-CoV-2 and SARS-CoV genes are most similar.^16^ A study of 74 SARS patients in the recovery phase of the 2002 SARS outbreak revealed the presence of antibodies in the plasma of all patients.^17^ IgG antibodies persisted at a detectable level for 720 days after infection. Importantly, NAbs persisted until day 720 in most patients.^17^ Another study of 56 SARS patients at the time of recovery demonstrated that the titers of IgG and neutralizing antibodies peaked at month 4 and diminished thereafter. IgG and neutralizing antibodies were undetectable in 19.4% and 11.1% of serum samples, respectively, at month 30, and in 25.8% and 16.1%, respectively, at month 36.^3^ Another study found that 56% of convalescent patients were positive 3 years after infection, and antibody levels dropped significantly 3 years after infection.^18^ These results suggest that NAbs at 2 weeks after infection are associated with the duration of immunity, and that these antibodies are still present 2 years after infection with SARS-CoV. During the COVID-19 outbreak, Seitz-Polski group found that the proportion of patients with positive SARS-CoV-2-specific IgA and IgG at admission was 9/13 (69%) and 6/13 (46%), respectively, and reached 100% for the two isotypes after 15 days of hospitalization. In the first 2 weeks after the admission for IgA and 4 weeks after the admission for IgG, titers for SARS-CoV-2 antibodies were generally increasing. The IgA level then decreased, although it was still positive even at 7 weeks, while that of IgG remained relatively stable over time.^19^ Lv group found an interesting observation in a 26-year-old female patient. Antiviral IgM was shown to be negative on day 56, day 68, and day 80 post disease onset. Antiviral IgG titers dropped from 46.69 on day 56 to 11.90 AU/ml on day 68, and were negative on day 80 after the onset of the symptoms, indicating disappearance of antibodies to SARS-CoV-2.^20^ In the current study, we used a chemiluminescent immunoassay (CLIA) sensitized with the SARS-CoV nucleoprotein (N) protein, receptor-binding domain (RBD) of the Spike (S) protein, S1 (N-terminal ectodomain containing the RBD), and N-S1 to detected the antibodies in the convalescent patients. While some of the recovered people had no antibodies, other convalescent patients were still positive for NP-, RBD-, S1-, and NP-S1-specific IgA, IgM, and IgG antibodies from 4 to 11 months after the onset of illness. This study described the longitudinal distribution of anti-SARS-CoV-2 antibodies in COVID-19 patients at 11 months after the onset. Moreover, we observed NAbs in these recovered patients. A total of 71.4% (60/84) of patients had positive NAbs at 9–11 months after the onset of illness. Not surprisingly, NAb was significantly correlated with anti-S1 and ant-RBD IgG, but not with anti-NP IgG. Anti-S1 or anti-RBD IgG can be used to analyze the serum neutralization ability in COVID-19 patients. Our results are consistent with the findings of other researchers,^14,15^ in terms of the humoral immunity’s role in blocking receptor binding as viruses enter host cells. This is an important observation because the presence of these antibodies may be necessary for the rehabilitation of patients and for the prevention of reinfection of SARS-CoV-2.

T cell response is an emerging key determinant for the control of SARS-CoV-2 infection.^21^ In a number of studies, the reduction in the number of T cells has been associated with poor clinical outcomes and immune pathogenesis, and adequate T cell counts and appropriate effector function are common in patients with mild disease symptoms or successful recovery.^22^ Thevarajan et al. followed up a 47-year-old female patient and found a concomitant increase in CD4+, CD8+, and antibody-secreting B cells from day 7 after infection, which persisted for 7 days as the symptoms disappeared.^23^ Other research have shown similar revival of the T cell response in recovered patients.^24^ Modulation and control of B cell response is the key to an effective immune response to coronavirus. The B cell subsets were significantly lower in patients with severe disease than in healthy controls. A recent study showed a similar decline in the number of NK cells and an increase in the expression of exhaustion markers in severe cases.^25^ On the contrary, another study found no significant difference in the total number of NK cells between non-ICU and ICU patients.^26^ This quantitative difference may be due to differences in the timing of immune responses and the underlying epidemiological disease conditions. Immune cell analysis data from the early recovery stage (ERS) and late recovery stage (LRS) showed that patients with COVID-19 had biphasic effects, with fewer NK cells in ERS, while NK cells recovered during LRS.^27^ Consistent with the results, we found that T, B, and NK cells recovered in LRS. Although there was no significant difference in cell counts between the groups during follow-up, our studies confirmed that a significantly higher proportion of COVID-19 convalescent individuals presented with reduced CD3+ CD8+ cell count, B cell count, and NK cell count.

A high inflammatory state was mediated by cytokines. However, the relative contribution of immune cells to pro-inflammatory molecules has continued to emerge during COVID-19, and published studies have shown complex interactions. It was demonstrated that the elevated serum levels of IL-2, IL-6, IL-10, and IFN-γ were associated with disease severity in a relatively similar sample size (*n* = 40).^21^ In a longitudinal analysis, the levels of IL-6 and IL-10 in severe cases were consistently increased (*n* = 13).^21^ In a relatively larger cohort, levels of IL-2R, IL-6, IL-8, IL-10, and TNF-α in patients who died of disease (*n* = 113) were higher compared with recovered patients (*n* = 161).^28^ Overall, all these studies point to an increase in the secretion of pro-inflammatory molecules in COVID-19. In our study, we found that most of the cytokines recovered in the convalescent individuals except that a significantly higher proportion of COVID-19 convalescent individuals presented with elevated IL-5, IL-6, and IL-1β levels. Interestingly, in addition to the correlation between B cell count and anti-NP and anti-NP-S IgG, the correlation between IL-6 and NAb, anti-NP IgG, and NP-S1 IgG, and the correlation between IL-1β and NAb were also noted.

We also study the cytokine response to SARS-CoV-2 antigen in healthy and COVID-19 convalescent patients. The recombinant antigen peptide pools induced strong immune responses by increasing IL-1β, IL-6, IL-8, IL-10, and TNF-α levels in both healthy donors and patients. Of note, compared with healthy donors, the concentrations of antigen peptide pools induced-IL-2 and -IFN-γ in patients were much higher, suggesting that IL-2 and IFN-γ had induced SARS-CoV-2-specific responses. The predictive value of the antigen peptide pools stimulated-IL-2 and -IFN-γ for COVID-19 convalescent individuals was also confirmed. Similar to our findings, studies from Sweden and Germany observed T cell responses against SARS-CoV-2 in convalescent persons,^29,30^ Sekine et al. reported that 4/31 (13%) patients who recovered from mild symptoms of COVID-19 were seronegative,^29^ which is similar to 29% of seronegative results in our cohort. Our data suggested that antigen peptide pools stimulated-IL-2 and -IFN-γ responses could distinguish COVID-19 convalescent individuals from healthy donors. Cytokine responses after antigen peptide pools stimulations were found in few ‘no COVID-19’ -individuals. These data are in agreement with previous studies demonstrating a cross-reactivity of SARS-CoV-2 antigens in ‘no COVID-19’ -individuals induced by a past exposure to seasonal cold coronaviruses.^31–34^ Therefore, a more species-specific peptide selection is needed to fully understand the immune response to SARS-CoV-2.

Interestingly, our data further indicated that NAb survival was significantly affected by the antigen peptide pools stimulated-IL-2 response, -IL-8 response, and -IFN-γ response. Antigen peptide pools stimulated-IL-2 and -IFN-γ positive responses were associated with significantly better survival of NAb, anti-S1 IgG, anti-RBD IgG, and anti-NP-S1 IgG in COVID-19 convalescent individuals, while the antigen peptide pools stimulated-IL-8 positive response was associated with significantly worse antibody survival.

CD4+ T and CD8+ T cells are the centers of antiviral response in COVID-19 patients. Antigen-specific CD4+ and CD8+ T cells have been found in rehabilitative patients.^35^ Braun’s group investigated a group of 18 COVID-19 patients and found reactive CD4+ cells (83%) in the blood from convalescent COVID-19 patients, which were specifically targeting the S protein.^24^ Meanwhile, another study found specific CD4+ T cells (100%) and CD8+ T cells (70%) in convalescent patients.^31^ Consistent with these studies, our study further confirmed the activation of CD4+ T, CD8+ T, and NK cells by stimulating the production of IFN-γ in vitro. Our data showed that PMA/ionomycin can induce simultaneous secretion of IFN-γ by CD4+, CD8+ T cells, and NK cells. After antigen stimulation, the activated status of CD8+ T cell counts was correlated with NAb. However, CD4+ T counts were not statistically significant correlated with NAb. The data suggest that the overall T cell response is heterogenous. Interestingly, SARS-CoV-2 directed NK cells showed a relatively low response in convalescent patients, but it did correlate with the NAb and disease severity. The limitation of our study is that the sample size of severe patients was very small. Our data suggest that the development of NAbs may be associated with the activation of antiviral T cells and NK cells. In addition, effective elimination of the virus may require a synergistic humoral and cellular immune response.

Several previous studies detected SARS-CoV-2-specific humoral and cellular immunity in discharged patients. Follow-up analysis on a cohort of six patients 2 weeks post discharge also revealed high titers of immunoglobulin G (IgG) antibodies.^14^ Another research enrolled 15 convalescent subjects with a follow-up of 25–56 days.^36^ Professor Buggert’s study revealed a strong positive correlation between IgG responses directed against the spike protein of SARS-CoV-2 and IgG responses directed against the nucleocapsid protein of SARS-CoV-2 in 66 convalescent individuals.^29^ Compared with these published research, our research included a larger cohort of COVID-19 patients and longer follow-up. For SARS-CoV-2, the focus is mainly on IgM, IgG, and IgA antibodies that can neutralise the virus by binding to the spike and other membrane proteins and thus preventing infection. We detect 12 kinds of antibodies, and 12 kinds of cytokines in response to SARS-CoV-2 peptide pools. Another novel conclusion in our study is that we first reported that NAb survival was significantly affected by the antigen peptide pools-stimulated-IL-2 response, -IL-8 response, and -IFN-γ response. Our study demonstrates a comprehensive understanding of the immunopathology of COVID-19 and the sustainability of protective immunity. Our results provide strong evidence that SARS-CoV-2 antibodies persist up to 11 months after symptom onset. We have also highlighted some of the immune responses that are critical to the progress and outcomes of COVID-19 patients. The findings of this study are important for assessing the risk of reinfection in previously exposed populations and the duration of antibody-mediated immunity provided by any candidate vaccine.

## Materials and methods

### Patients

This study was carried out according to the suggestion of the Ethics Committee of Zhongnan Hospital of Wuhan University. Blood samples from both COVID-19 convalescent individuals and healthy donors were taken from Zhongnan Hospital of Wuhan University. This study was approved by the Ethics Committee of Zhongnan Hospital of Wuhan University. Study inclusion criteria included subjects with a clinical and/or laboratory diagnosis of COVID-19 over the age of 20 years, regardless of disease severity, gender, pregnancy or nursing status, or the presence of other medical conditions, who were willing and able to provide informed consent. Study exclusion criteria included lack of willingness or ability to provide informed consent. Subjects could be excluded if blood donation was deemed to be medically unsafe or otherwise not in the best medical interest of the subject.

### Analysis of anti-SARS-CoV-2 antibodies

The SARS-CoV-2 NAb assay (SHENZHEN YHLO BIOTECH CO., LTD, Shenzhen, China, Cat#C86109) is a paramagnetic particle chemiluminescent immunoassay (CLIA) for qualitative detection of SARS-CoV-2 NAb in human serum and plasma using the automated iFlash immunoassay system. It is mainly used for the evaluation of NAbs in patients recovering from COVID-19 or the auxiliary evaluation of the effect of the SARS-CoV-2 vaccine.

The iFlash-SARS-CoV-2 IgA/IgG/IgM assay is a paramagnetic particle chemiluminescent immunoassay (CLIA) for qualitative determination of the IgG antibody to SARS-CoV-2 in human serum or plasma using the iFlash immunoassay system. The iFlash-SARS-CoV-2 IgA/IgG/IgM aids in the diagnosis of SARS-CoV-2 infection and the determination of immunity. The manufacturer has determined a cut-off value of 10.00 AU/mL for the antibodies.

### Analysis of lymphocyte subpopulations

The BD Multitest 6-color TBNK reagent (Cat# 644611) contains the following antibodies to identify and enumerate the different lymphocyte subgroups (Supplemental Fig. 1): CD3 FITC for the identification of T lymphocytes, CD16 and CD56 PE for identifying NK lymphocytes, CD45 PerCP-Cy™5.5 to allow for gating on the lymphocyte populations, CD4 PE-Cy™7 for detecting T helper/inducer lymphocytes, CD19 APC to identify B lymphocytes, and CD8 APC-Cy7 for the identification of the suppressor/cytotoxic T lymphocyte subset. We pipetted 20 μL of BD Multitest 6-color TBNK reagent into the bottom of the BD Trucount tube and then pipetted 50 μL of well-mixed, anticoagulated whole blood into the bottom of the tube. The tube was capped and vortexed gently to mix, followed by incubation for 15 min in the dark at room temperature. We added 450 μL of 1X BD FACS lysing solution to the tube and incubated the tube for 15 min in the dark at room temperature. Lymphocyte subpopulations were acquired and analyzed with BD FACSCanto clinical software.

### Cytokine analysis

This method involved multiplex cytometric bead array (CBA) for quantitative analysis of 12 kinds of cytokines, including IL-1β, IL-2, IL-4, IL-5, IL-6, IL-8, IL-10, IL-12, IL-17, interferon-gamma (IFN-γ), tumor necrosis factor-alpha (TNF-α), and IFN-α. The multiplex CBA was performed according to the manufacturer’s instructions. Briefly, 25 μL serum was mixed with equal volumes of capture beads and incubated with 25 μL of PE-conjugated antibodies for 2.5 h at room temperature in the dark. Then, the beads were centrifuged at 200 g for 5 min, and the supernatant was gently aspirated and resuspended in phosphate-buffered saline (PBS) (100 μL). The CBA was addressed in a FACS flow cytometer (BD Bioscience-Pharmingen), and it was analyzed using clinical software. Concentrations above the detection range (5000 pg/ml) were converted to the highest value of the standard curve.

### IFN-γ secretion assay

The level of IFN-γ secretion by CD4+, CD8+ T cells, and NK cells was measured in whole blood using the following procedures: (1) 100 μl of whole blood was diluted with 400 μl of IMDM medium (Gibico-BRL) and then it was stimulated with Leukocyte Activation Cocktail (BD GolgiPlug™, including 50 ng/ml PMA, 1 μM ionomycin, and 1 μg/ml brefeldin A) or SARS-CoV-2 antigen (0.03 μg/ml RBD, 0.03 μg/ml S1, 0.03 μg/ml S2, and 0.015 μg/ml N) for 4 h at 37 °C with 5% CO_2_. (2) After stimulation, 200 μl of the supernatant was extracted and incubated at room temperature for 15 min with 5 kinds of antibodies (anti-CD45-APC/H7, anti-CD3-FITC, anti-CD4-V450, anti-CD56-PE/Cy7, and anti-CD8-APC) (BD Biosciences). (3) After the red blood cells were lysed, the cell suspension was fixed and permeated with the Fixation/Permeabilization Buffer at room temperature for 15 min. (4) After washing, the cell suspensions were stained with anti-IFN-γ-PE (BD Pharmingen) at room temperature for 15 min. (5) After washing, the cell pellets were resuspended in 200 μl PBS and analyzed using a flow cytometer (BD Bioscience-Pharmingen).

### Statistical analysis

Statistical analysis was performed using SPSS (Version 22.0, SPSS, Inc., Chicago, IL, USA). Statistical analysis of the results was performed using the Student’s *t* test when only two groups were compared, or one-way analysis of variance when more than two groups were compared. Pearson’s correlation coefficients were calculated. The descriptive statistics included frequency analysis (percentages) of the categorical variables. With respect to the laboratory results, we assessed whether the measurements were within the normal range. Non-parametric tests were used if the data were not distributed normally according to the Shapiro–Wilk normality test. Receiver-operator characteristic (ROC) analysis for evaluating diagnostic performance; We used SPSS Version 22.0 to manage and analyze the data and performed a survival analysis for interval-censored data to estimate the duration of SARS-CoV-2 antibodies detection. For this procedure, we considered survival to be the detection of SARS-CoV-2 antibodies detection (a positive result). *P* values <0.05 were considered statistically significant.

## Supplementary information

SUPPLEMENTAL MATERIAL

## Data Availability

The datasets used and/or analyzed to support the findings of this study are available in this paper or the Supplementary Information. Any other raw data that support the findings of this study are available from the corresponding author upon reasonable request.
