# Supplementary material for: SARS-CoV-2-specific immune response in COVID-19 convalescent individuals
Source: Signal Transduct Target Ther. 2021 Jul 7;6:256. doi: 10.1038/s41392-021-00686-1 (PMC8261819; doi:10.1038/s41392-021-00686-1)
Supplement: Supplementary file 1 [file 41392_2021_686_MOESM1_ESM.pdf]

Supplementary Materials for

SARS-CoV-2-specific immune response in COVID-19 convalescent individuals

Yunbao Pan, Xianghu Jiang, Liu Yang, Liangjun Chen, Xiaojiao Zeng, Guohong Liu, Yueting Tang, Chungen Qian, Xinming Wang, Fangming Cheng, Jun Lin, Xinghuan Wang, Yirong Li

Correspondence to: liyirong2021@126.com

This PDF file includes:

Figures. S1 to S3
Tables S1 to S3

Figure. S1. The flow cytometry analysis template to detect the CD3+, CD3+CD4+, CD3+CD8+, CD19+, and CD16+CD56+ cells in one tube simultaneously.

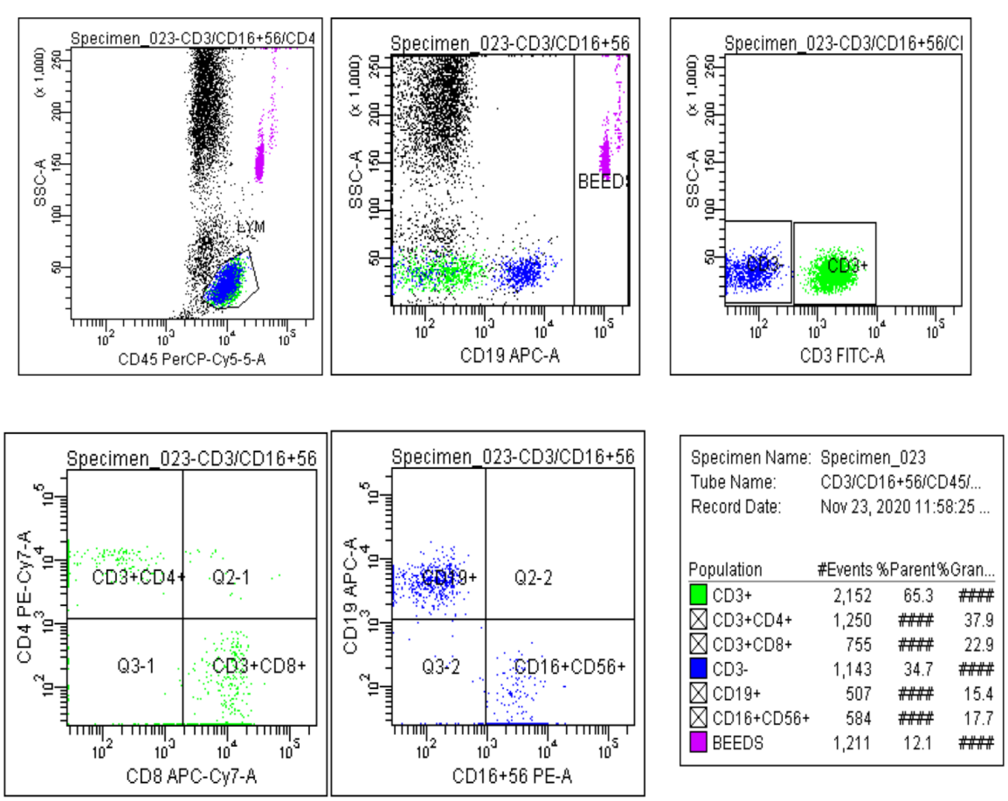

Figure. S2.

(a) The flow cytometry analysis template to detect 12 kinds of cytokines in one tube simultaneously. (B) The expression of cytokines in COVID-19 convalescent individuals. *P<0.05, 0.05<**P<0.001, ***P<0.001.

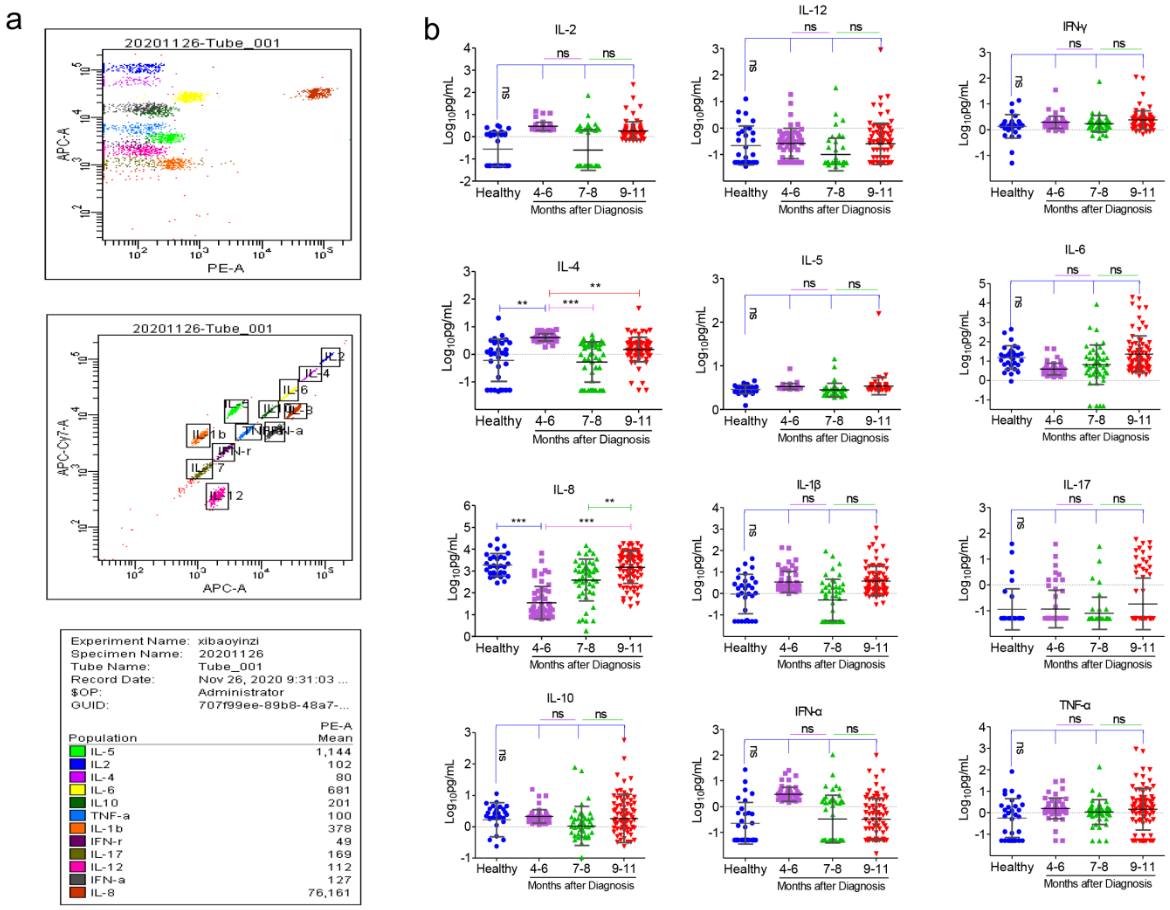

Figure. S3.

The flow cytometry analysis template to detect intracellular IFN-γ (a) and IgG isotype control (b).

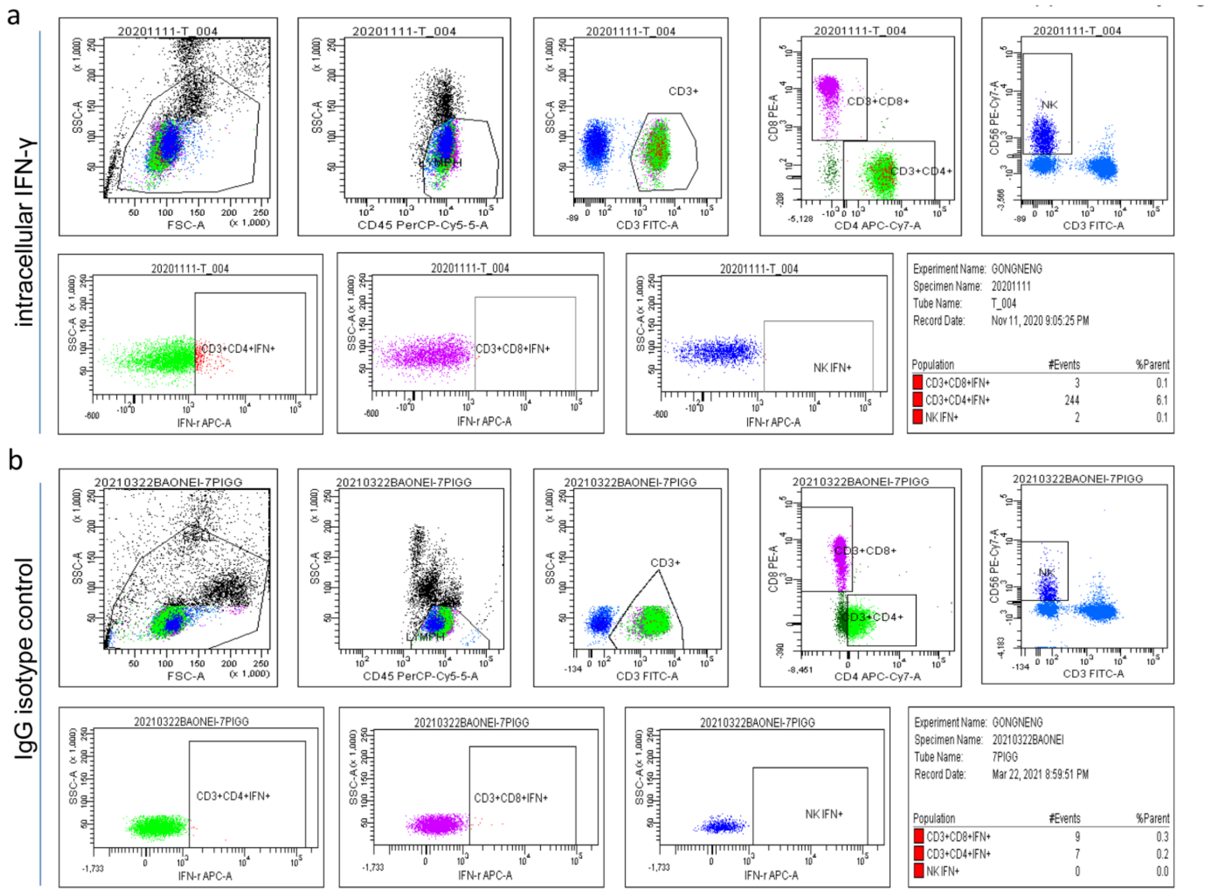

Table S1. Immunological and pathological characterization of COVID-19 convalescent individuals

Variate		4-6 months N=47	7-8 months N=66	9-11 months N=84	P
Sex	male	17	37	36	0.089
	female	30	29	48	
Age	mean	41	58	45	0.000
Hospitalization status					
	Hospitalized at the time of blood collection (%)	0	0	0	
Sample Collection Dates		July 15, 2020 -July 31, 2020	Sep 7, 2020 -Sep 23, 2020	Nov 11, 2020 -Dec 10, 2020	
Past Medical History					
	Hyperlipidemia (%)	3(6%)	9(14%)	2(2%)	
	Hyperglycemia(%)	3(6%)	15(23%)	1(1%)	
	Chronic infection (%)	1(0.2%)	2(0.3%)	2(0.2%)	
	Connective tissue disease (%)	0	0	0	
CD3+%	High	48.94%	24.24%	29.79%	0.002
	Normal	51.06%	74.24%	59.52%	
	Low	0%	1.52%	10.71%	
CD3+Abs Cnt	High	0%	0%	0%	0.018
	Normal	95.74%	92.42%	80.95%	
	Low	4.26%	7.58%	19.05%	
CD3+CD4+%	High	42.55%	37.88%	41.67%	0.789
	Normal	57.45%	62.12%	57.14%	
	Low	0%	0%	1.19%	
CD3+CD4+Abs	High	0%	0%	0%	0.030
	Normal	95.74%	100%	90.48%	
	Low	4.26%	0%	9.52%	
CD3+CD8+%	High	8.51%	6.06%	5.95%	0.044
	Normal	87.23%	89.39%	76.19%	
	Low	4.26%	4.55%	17.86%	
CD3+CD8+Abs	High	0%	0%	0%	0.027
	Normal	76.6%	74.24%	57.14%	
	Low	23.4%	25.76%	42.86%	

4/8 Ratio	High	21.28%	18.18%	35.71%	0.062
	Normal	63.83%	59.09%	42.86%	
	Low	14.89%	22.73%	21.43%	
CD19+%	High	0%	0%	3.57%	0.038
	Normal	59.57%	36.36%	45.24%	
	Low	40.43%	63.64%	51.19%	
CD19+Abs	High	0%	0%	0%	0.614
	Normal	44.68%	36.36%	36.9%	
	Low	55.32%	63.64%	63.1%	
CD16+CD56+%	High	4.26%	19.7%	7.14%	0.002
	Normal	76.6%	78.79%	82.14%	
	Low	19.15%	1.52%	10.71%	
CD16+CD56+Abs	High	0%	1.52%	0%	0.004
	Normal	72.34%	90.91%	71.43%	
	Low	27.66%	7.58%	28.57%	
WBC	High	2.13%	1.52%	1.19%	0.522
	Normal	89.36%	96.97%	92.86%	
	Low	8.51%	1.52%	5.95%	
RBC	High	0%	3.03%	1.19%	0.675
	Normal	70.21%	72.73%	75%	
	Low	29.79%	24.24%	23.81%	
PLT	High	2.13%	0%	4.76%	0.275
	Normal	97.87%	96.97%	91.67%	
	Low	0%	3.03%	3.57%	
NEUT	High	2.13%	1.52%	1.19%	0.626
	Normal	82.98%	92.42%	88.1%	
	Low	14.89%	6.06%	10.71%	
LYMPH	High	0%	18.18%	3.57%	0.034
	Normal	80.85%	81.82%	91.67%	
	Low	19.15%	0%	4.76%	
MONO	High	29.79%	18.18%	3.57%	0.001
	Normal	70.21%	81.82%	95.24%	
	Low	0%	0%	1.19%	
EO	High	2.13%	1.52%	1.19%	0.072
	Normal	65.96%	81.82%	86.9%	
	Low	31.91%	16.67%	11.9%	
BASO	High	12.77%	18.18%	9.52%	0.000
	Normal	72.34%	7.58%	61.9%	

	Low	14.89%	74.24%	28.57%	
ALT	High	12.77%	6.06%	10.71%	0.447
	Normal	87.23%	93.94%	89.29%	
	Low	0%	0%	0%	
AST	High	0%	0%	0%	
	Normal	100%	100%	100%	---
	Low	0%	0%	0%	

Abbreviation: CD3+Abs Cnt, CD3+ absolute cell count; CD3+CD4+Abs Cnt, CD3+CD4+ absolute cell count; CD3+CD8+Abs Cnt, CD3+CD8+ absolute cell count; CD19+Abs Cnt, CD19+ absolute cell count; CD16+CD56+Abs Cnt, CD16+CD56+ absolute cell count; WBC, white cell count; RBC, red blood cell count; PLT, platelet; NEU, neutrophile; LYM, lymphocyte; MONO, monocyte; EO, eosinophile; BASO, basophile; ALT, alanine aminotransferase; AST, aspartate aminotransferase.

Table S2. Cytokines of COVID-19 convalescent individuals

Variate		4-6 months N=55	7-8 months N=47	9-11 months N=81	P
Sex	male	20	28	36	0.060
	female	35	19	45	
Age	mean	41	51	46	0.000
IL-2	High	5.45%	2.13%	4.94%	0.676
	Normal	94.55%	97.87%	95.06%	
IL-4	High	0%	0%	1.23%	0.531
	Normal	100%	100%	98.77%	
IL-5	High	36.36%	6.38%	24.69%	0.002
	Normal	63.64%	93.62%	75.31%	
IL-6	High	3.64%	23.4%	38.27%	0.000
	Normal	96.36%	76.6%	61.73%	
IL-8	High	40%	12.77%	0%	0.000
	Normal	60%	87.23%	100%	
IL-1β	High	16.36%	8.51%	19.75%	0.242
	Normal	83.64%	91.49%	80.25%	
IL-17	High	1.82%	2.13%	7.41%	0.201
	Normal	98.18%	97.87%	92.59%	
IL-10	High	5.45%	6.38%	22.22%	0.005
	Normal	94.55%	93.62%	77.78%	
IFN-α	High	9.09%	4.62%	4.94%	0.510
	Normal	90.91%	95.74%	95.06%	
TNF-α	High	9.09%	6.38%	19.75%	0.055
	Normal	90.91%	93.62%	80.25%	
IL-12	High	3.64%	4.26%	8.64%	0.407
	Normal	96.36%	95.74%	91.36%	
IFN-γ	High	1.82%	2.13%	3.7%	0.769
	Normal	98.18%	97.87%	96.3%	

Table S3. Cox proportional hazards regression analysis for antibodies survival

Cytokine	nCoV-2-NAb		Anti-NP IgG		Anti-S1 IgG		Anti-RBD IgG		Anti-NP-S1 IgG	
	P	HR (95% CI)	P	HR (95% CI)	P	HR (95% CI)	P	HR (95% CI)	P	HR (95% CI)
IL-2	.603	0.77 (0.2-2.0)	.290	0.6 (0.2-1.4)	.021	0.1 (0.03-0.7)	.210	0.4 (0.1-1.5)	.012	0.1 (0.01-0.6)
IL-4	.748	0.61 (0.031-12.2)	.185	0.2 (0.02-2.1)	.399	23.2 (0.01-34368.0)	.838	1.3 (0.05-33.0)	.990	0 (0-.)
IL-5	.865	1.11 (0.3-3.9)	.661	1.1 (0.5-2.6)	.459	2.1 (0.2-17.1)	.967	1.0 (0.2-5.0)	.341	3.1 (0.3-32.4)
IL-6	.995	18273.8 (0-.)	.983	5476.9 (0-.)	.997	64908.5 (0-.)	.996	54891.4 (0-.)	1.000	1.209 (0-.)
IL-8	.001	9.4 (2.5-34.4)	.000	6.5 (2.7-15.6)	.018	14.4 (1.5-132.5)	.013	5.9 (1.4-24.1)	.945	1259021.5 (0-3.9E+178)
IL-17	.306	2.4 (0.4-14.4)	.660	1.3 (0.3-4.9)	.545	0.2 (0.002-26.8)	.789	1.3 (0.1-12.0)	.854	0.5 (0.001-267.9)
IL-10	1.00	0.432 (0-.)	.968	0 (0-5.4E+209)	1.00	5.3 (0-.)	1.00	1.2 (0-.)	.999	215.7 (0-.)
IFN-α	.610	0.4 (0.03-7.2)	.662	1.5 (0.2-9.9)	.869	0.5 (0-757.6)	.885	1.2 (0.06-25.9)	.407	13.2 (0.03-5883.2)
TNF-α	.998	0.001 (0-.)	.993	39.6 (0-.)	.999	0.002 (0-.)	.998	0 (0-.)	1.000	3.2 (0-.)
IL-12	.613	1.4 (0.3-5.3)	.817	0.8 (0.2-2.8)	.779	0.7 (0.1-5.4)	.869	1.1 (0.2-5.4)	.764	0.7 (0.09-5.6)
IFN-γ	.005	0.16 (0.04-0.5)	.359	0.6 (0.2-1.6)	.181	0.2 (0.02-1.9)	.026	0.1 (0.03-0.8)	.301	0.2 (0.02-3.2)
IL-1β	.992	269821.7 (0-.)	.970	12585.6 (0-1.4E+218)	.996	27935.4 (0-.)	.994	249932.3 (0-.)	.996	16710.7 (0-.)

HR: Hazard Ratio
